# Supplementary material for: A neuropeptide regulates fighting behavior in Drosophila melanogaster
Source: eLife. 2020 Apr 21;9:e54229. doi: 10.7554/eLife.54229 (PMC7173970; doi:10.7554/eLife.54229)
Supplement: Supplementary file 1. [file elife-54229-supp1.docx]

| **Key Resources Table** | | | | |
| --- | --- | --- | --- | --- |
| **Reagent type (species) or resource** | **Designation** | **Source or reference** | **Identifiers** | **Additional information** |
| antibody | Mouse anti-Bruchpilot antibody (nc82) | Developmental Studies Hybridoma Bank | Cat# nc82, RRID:AB_2314866 | IHC (1:50) |
| antibody | HA-Tag Rabbit mAb | Cell Signaling Technologies | Cat# 11846, RRID:AB_2665471 | IHC (1:300) |
| antibody | mAb anti-DYKDDDDK Epitope Tag [L5] | Novus Biologicals | Cat# NBP1-06712, RRID:AB_1625981 | IHC (1:200) |
| antibody | Rat monoclonal anti-HA | Roche | Cat# 11867431001, RRID:AB_390919 | IHC (1:100) |
| antibody | mouse monoclonal anti-GFP-20 | Sigma-Aldrich | Cat# G6539, RRID:AB_259941 | IHC (1:100) |
| antibody | Rabbit polyclonal anti-GFP | Thermo Fisher Scientific | Cat# A-11122, RRID:AB_221569 | IHC (1:1000) |
| antibody | chicken polyclonal anti-GFP | Thermo Fisher Scientific | Cat# A10262, RRID:AB_2534023 | IHC (1:1000) |
| antibody | Goat anti-rabbit, Alexa Fluor 488 | Thermo Fisher Scientific | Cat# A-11034, RRID:AB_2576217 | IHC (1:500) |
| antibody | Goat anti-chickent, Alexa Fluor 488 | Thermo Fisher Scientific | Cat#A-11039; RRID: AB_2534096 | IHC (1:500) |
| antibody | Goat anti-mouse, Alexa Fluor 546 | Thermo Fisher Scientific | Cat# A-11030, RRID:AB_2534089 | IHC (1:500) |
| antibody | Goat anti-rat, Alexa Fluor 546 | Thermo Fisher Scientific | Cat# A-11081, RRID:AB_2534125 | IHC (1:500) |
| antibody | Goat anti-rabbit, Alexa Fluor 546 | Thermo Fisher Scientific | Cat# A-11010, RRID:AB_2534077 | IHC (1:500) |
| antibody | Goat anti-rat, Alexa Fluor 633 | Thermo Fisher Scientific | Cat# A-21094, RRID:AB_2535749 | IHC (1:500) |
| antibody | Rabbit polyclonal anti-RFP | Thermo Fisher Scientific | Cat# R10367, RRID:AB_10563941 | IHC (1:500) |
| antibody | Goat anti-rat, Alexa Fluor 647 | Thermo Fisher Scientific | Cat# A-21247, RRID:AB_141778 | IHC (1:500) |
| antibody | Rabbit polyclonal anti-DSK |  | N/A | IHC(1:1000) |
| chemical compound, drug | Paraformaldehyde (PFA) | Electron Microscopy Sciences | Cat# 15713 | 8% PFA diluted in 1XPBS at 1:4 or 1:2 |
| chemical compound, drug | DPX Mountant | Sigma-  Aldrich | Cat# 44581 |  |

| chemical compound, drug | Normal goat serum | Sigma-  Aldrich | Cat# G9023 |  |
| --- | --- | --- | --- | --- |
| chemical compound, drug | Adenosine 5’-triphosphate disodium salt hydrate microbial | Sigma-  Aldrich | Cat# A6419-1G | 2.5mM |
| chemical compound, drug | Mifepristone (RU486) | Sigma-  Aldrich | Cat# M8046-1G |  |
| Genetic reagent  (*D.melanogaster)* | *UAS-myrGFP,QUAS-mtdTomato(3*HA);trans-Tango* | Zhong Lab, Tsinghua University | N/A |  |
| Genetic reagent  (*D.melanogaster)* | +*; sp/cyo; LexAop-P2X2, UAS-GCamP/Tm2* | Luo Lab, Peking University | N/A |  |
| Genetic reagent  (*D.melanogaster)* | *+; sp/cyo; UAS-P2X2, UAS-GCamP/Tm2* | Luo Lab, Peking University | N/A |  |
| Genetic reagent  (*D.melanogaster)* | *+; sp/cyo; LexAOP-GCAMP6, UAS-P2X2/Tm2* | Luo Lab, Peking University | N/A |  |
| Genetic reagent  (*D.melanogaster)* | *LexAop2-mCD8::GFP* | Bloomington Stock Center | # 32203 |  |
| Genetic reagent  (*D.melanogaster)* | *UAS-mCD8::GFP* | Bloomington Stock Center | # 5137 |  |

| Genetic reagent  (*D.melanogaster)* | *10XUAS-IVS-mCD8::RFP,13XLexAop2-mCD8::GFP; nSyb-MKII::nlsLexADBD/CyO; UAS-p65AD::CaM* | Bloomington Stock Center | # 61679 |  |
| --- | --- | --- | --- | --- |
| Genetic reagent  (*D.melanogaster)* | *y[1] v[1]; P{y[+t7.7] v[+t1.8]=TRiP.JF02644}attP2* | Bloomington Stock Center | # 27494 |  |
| Genetic reagent  (*D.melanogaster)* | *R15A01-p65.AD* | Bloomington Stock Center | # 68837 |  |
| Genetic reagent  (*D.melanogaster)* | *R71G01-GAL4.DBD* | Bloomington Stock Center | # 69507 |  |
| Genetic reagent  (*D.melanogaster)* | *R71G01-LexA* | Bloomington Stock Center | # 54733 |  |
| Genetic reagent  (*D.melanogaster)* | *R57C10-Flp2::PEST in su(Hw)attP8;; HA_V5_FLAG (MCFO)* | Bloomington Stock Center | # 64089 |  |
| Genetic reagent  (*D.melanogaster)* | +; UAS-syteGFP, UAS-Denmark; Sb/+ | Bloomington Stock Center | # 33604 |  |
| Genetic reagent  (*D.melanogaster)* | *UAS-myrRFP* | Li Lab, Peking University | N/A |  |
| Genetic reagent  (*D.melanogaster)* | UAS>stop>Kir2.1^eGFP^ | Rao Lab, Peking University | N/A |  |
| Genetic reagent  (*D.melanogaster)* | hs-FLP | Pan Lab, Southeast University | N/A |  |
| Genetic reagent  (*D.melanogaster)* | elav-GS | Zhong Lab, Tsinghua University | N/A |  |
| Genetic reagent  (*D.melanogaster)* | *UAS-dTrpA1/cyo* | Garrity Lab, Brandeis University | N/A |  |
| Genetic reagent  (*D.melanogaster)* | *UAS-TNT* | O'Kane Lab, University of Cambridge | N/A |  |
| Genetic reagent  (*D.melanogaster)* | *UAS-impTNT* | O'Kane Lab, University of Cambridge | N/A |  |
| Genetic reagent  (*D.melanogaster)* | *UAS-Kir2.1* | Bloomington Stock Center | #6595、#6596 |  |
| Genetic reagent  (*D.melanogaster)* | *Dsk*^GAL4^ | Rao Lab, Peking University | N/A |  |
| Genetic reagent  (*D.melanogaster)* | *ΔDsk*^GAL4^ | Zhou Lab, Chinese Academy of Sciences, this paper | N/A |  |
| Genetic reagent  (*D.melanogaster)* | *CCKLR-17D1*^GAL4^ | Rao Lab, Peking University | N/A |  |
| Genetic reagent  (*D.melanogaster)* | *CCKLR-17D3*^GAL4^ | Rao Lab, Peking University | N/A |  |
| Genetic reagent  (*D.melanogaster)* | *△Dsk* | Rao Lab, Peking University | N/A |  |
| Genetic reagent  (*D.melanogaster)* | *△CCKLR-17D1* | Rao Lab, Peking University | N/A |  |
| Genetic reagent  (*D.melanogaster)* | *△CCKLR-17D3* | Rao Lab, Peking University | N/A |  |
| Genetic reagent  (*D.melanogaster)* | *UAS-Dsk* | Zhou Lab, Chinese Academy of Sciences, this paper | N/A |  |
| Genetic reagent  (*D.melanogaster)* | *UAS-CCKLR-17D1* | Zhou Lab, Chinese Academy of Sciences, this paper | N/A |  |
| Genetic reagent  (*D.melanogaster)* | *elav-GAL4*;  *UAS-dcr2* | Rao Lab, Peking University | N/A |  |
| Genetic reagent  (*D.melanogaster)* | *Lexo-CD4-spGFP11/CyO*; *UAS-CD4-spGFP1-10/Tb* | Gordon et al., 2009 | N/A |  |
| recombinant DNA reagent | pBSK-attP-3P3-RFP-loxP | Bowen Deng et al., 2019 | N/A |  |
| recombinant DNA reagent | pBSK-attB-loxP-myc-T2A-Gal4-GMR-miniwhite | Bowen Deng et al., 2019 | N/A |  |
| recombinant DNA reagent | pBSK-attB-loxP-V5-T2A-LexA::p65-GMR-miniwhite | Bowen Deng et al., 2019 | N/A |  |
| software, algorithm | MATLAB | MathWorks, Natick, MA | <https://www.mathworks.com/products/matlab.html> |  |
| software, algorithm | ImageJ | National Institutes of Health | https://imagej.nih.gov/ij/ |  |
| software, algorithm | Prism 7 | GraphPad | <https://www.graphpad.com/> |  |
